# Development of an artificial intelligence based model for predicting the euploidy of blastocysts in PGT-A treatments

**DOI:** 10.1038/s41598-023-29319-z

**Published:** 2023-02-09

**Authors:** Zhenya Yuan, Mu Yuan, Xuemei Song, Xiaojie Huang, Weiqiao Yan

**Affiliations:** Reproductive Medicine Center, Xuzhou Maternal and Child Health Care Hospital, Xuzhou, 221009 China

**Keywords:** Diseases, Infertility

## Abstract

The euploidy of embryos is unpredictable before transfer in in vitro fertilisation (IVF) treatments without pre-implantation genetic testing (PGT). Previous studies have suggested that morphokinetic characteristics using an artificial intelligence (AI)-based model in the time-lapse monitoring (TLM) system were correlated with the outcomes of frozen embryo transfer (FET), but the predictive effectiveness of the model for euploidy remains to be perfected. In this study, we combined morphokinetic characteristics, morphological characteristics of blastocysts, and clinical parameters of patients to build a model to predict the euploidy of blastocysts and live births in PGT for aneuploidy treatments. The model was effective in predicting euploidy (AUC = 0.879) but was ineffective in predicting live birth after FET. These results provide a potential method for the selection of embryos for IVF treatments with non-PGT.

## Introduction

The appearance of two pronuclei (2PN) 16–18 h after fertilisation is called normal fertilisation. The genetic state of embryos is unpredictable before transfer in in vitro fertilization (IVF) treatments. Typically, normal fertilisation and embryos of high morphological grade are initially selected for transfer^1^. However, 2PN embryos can also have chromosomal abnormalities, which increase or decrease the number of whole chromosomes or fragments, resulting in infertility, recurrent pregnancy loss (RPL), or birth defects after embryo transfer^2^. Pre-implantation genetic testing (PGT) technology based on high-throughput sequencing or microarray can detect the genetic status of embryos before transfer and select euploid embryos for transfer to reduce the risk of pregnancy^3,4^. The most important yet difficult task in IVF treatment with non-PGT is the selection of euploid embryos. Previous studies have shown that the euploidy rate of embryos is correlated with female age, morphological grade, and the number of frozen days of embryos^5–7^. However, it is subjective, less rigorous and not sufficient to predict the euploidy of embryos based on these factors alone in non-PGT treatments.

Embryonic development is a complex and dynamic process. The conventional morphological grading method for embryos is limited to observation and evaluation at a certain point in the embryo development period when observed under a microscope. Furthermore, this method requires the embryos to be exposed to changing environments, which could affect their developmental potency. Frequently, embryos are ignored when 2PN or important morphological images are not observed under a microscope. Although PGT can show the genetic state of embryos before transfer, biopsies in PGT treatments may damage the developmental potency of embryos. In addition, PGT technology is strictly restricted in clinical application in China.

In recent years, the morphokinetic characteristics of embryos have been associated with the assessment of their developmental potency without invasive testing using the AI method. Meseguer et al. proved that embryo implantation can be predicted by an AI-based model using morphological and morphokinetic characteristics^8^. A preclinical validation study by Storr et al. suggested that the results of these studies may differ from clinical settings, algorithms, or IVF clinics^9^. In 2020, Ver Milyea et al. showed an effective and positive prediction of embryo viability using an AI-based model using static images captured by optical light microscopy in a multicentre study including 8,886 embryos and 11 IVF clinics^10^. Lee et al. demonstrated that euploid embryos possessed better morphokinetic expressions than mosaic embryos and proved that the generally applicable KIDScore D5 model was able to distinguish euploid blastocysts with specific morphokinetic characteristics^11^. Furthermore, AI-based model has been applied to predict FET outcomes in many studies, showing effective and positive performance. Adolfsson et al. proved that compared to the morphological grade (Gardner Schoolcraft criteria), the D3 KIDScore model was superior for D3 embryos and blastocysts in predicting live birth^12^. Petersen et al. found that the KIDScore model could predict the implantation potency of embryos transferred on D3^13^. Kato et al. demonstrated that the KIDScore model worked well for the prediction of pregnancy and live birth outcomes in patients of advanced age^14^. Ueno et al. demonstrated a deep learning model capable of predicting pregnancy after FET of blastocysts^15^. Gazzo et al. reported that embryo selection using the KIDScore model improved implantation rates after FET, and embryos with the highest KIDScore had a higher probability of being euploid and implanting^16^. The KIDScore model was significantly associated with implantation rates after FET in D5 blastocysts, although Reignier et al. reported that the predictive performance required further improvement^17^. Adolfsson et al. negated their previous study which proved that the KIDScore model was superior to the Gardner grade of blastocysts in predicting live births^12^. The authors concluded that the KIDScore seemed unsuccessful in showing better performance than the morphological grade of blastocysts, even when studied in a small sample size, but it was undeniable that the KIDScore was still effective in predicting live birth^18^. The above studies demonstrated the application value of AI-based model based on morphological and morphokinetic characteristics, providing a clinical basis and potential for developing further applications of AI-based models in IVF treatments.

One of the most important goals of IVF is to acquire as many euploid embryos as possible. The selection of euploid embryos for non-PGT treatment is challenging; however, they have excellent developmental potency and offer an extremely high chance of live birth. Better morphokinetic performance of embryos is more likely to be euploid. Studying the predictive potency of the AI-based model in the euploidy of embryos avoids the influence of endometrial conditions, subjective selection for embryos, and immunological factors after frozen embryo transfer (FET) in the analysis. Moreover, PGT technology could provide an objective assessment of the genetic status of embryos, help to study model to predict the euploidy of embryos in non-PGT treatments. Besides, TLM system enables continuous assessment during the embryonic developmental period and avoids defining this development, which is a dynamic process, by static observations; the morphokinetic characteristics of embryos provide a basis for predicting the euploidy of the embryos. Meanwhile, TLM can reduce the exposure time to changing environments. Although many study proved that AI-based model was effectively to predict the FET outcomes in IVF treatments, however, a previous study suggested that the results of studies may differ from clinical settings or IVF clinics^9^. In our study, we comprehensively assessed the predictive effect of female age, clinical indications of PGT (implantation failure (RIF), RPL, severe teratozoospermia (STS), and female advanced age (FAA: equal or older than 38 years old and require assisted reproductive technology)), number of embryonic frozen days, morphological characteristics (Gardner grade of blastocysts), morphokinetic characteristics (time to reach two cells (t2), three cells (t3), four cells (t4), five cells (t5), time to reach blastocyst (tB), KIDScore of blastocysts in D5) of the euploid blastocysts, and live birth after FET in TLM in PGT treatments, in order to provide a basis for embryo selection in non-PGT IVF treatments.

## Results

In this study, a total of 403 PGT for aneuploidy (PGT-A) treatments were investigated, including 83 patients with RIF, 184 patients with RPL, 83 patients with STS, and 53 patients with FAA (Table 1). There was no significant difference in female age among patients in the RIF, RPL, and STS groups (*P* > 0.05), but it was significantly lower than that in the FAA group (*P* < 0.05). A total of 1,396 blastocysts were successfully tested in PGT-A, including 296 in the RIF group, 691 in the RPL group, 274 in the STS group, and 135 in the FAA group. In the current study, the rates of euploidy, mosaicism, and aneuploidy in all PGT-A treatments were 52.51, 10.32, and 37.17%, respectively. There was no significant difference in the euploidy rate between the RIF, RPL, and STS groups (*P* > 0.05), but it was significantly higher than that in the FAA group (*P* < 0.05).

**Table 1:** Introduction of PGT-A treatments in this study.

Clinical indication of PGT-A	Female age (mean ± SD)	Number of PGT-A	Number of blastocysts	Euploidy rate (%)	Mosaic rate (%)	Aneuploidy rate (%)
RIF	35.01 ± 4.48	83	296	52.70 (156/296)	8.45 (25/296)	38.85 (115/296)
RPL	34.67 ± 4.68	184	691	53.84 (372/691)	9.84 (68/691)	36.32 (251/691)
STS	34.28 ± 4.49	83	274	57.30 (157/274)	11.68 (32/274)	31.02 (85/274)
FAA	40.85 ± 2.84	53	135	35.56 (48/135)	14.07 (19/135)	50.37 (68/135)
In total	35.47 ± 4.86	403	1396	52.51 (733/1396)	10.32 (144/1396)	37.17 (519/1396)

### Relationship between morphological characteristics and euploidy of blastocysts

In the present study, 773 and 623 blastocysts were frozen on D5 and D6, respectively (Table 2). The euploidy rate of D5 blastocysts graded as AA in patients < 38 years old was 98.43%, and in patients ≥ 38 years of age was 70.83%, significantly higher than that of D5 blastocysts graded as AB, BA, BB, BC, or CB (*P* < 0.05). The euploidy rate of D6 blastocysts graded as AA in patients younger than 38 years was 96.49%, and in patients ≥ 38 years of age was 88.24%, significantly higher than that of D6 blastocysts graded as AB, BA, BB, BC, or CB (*P* < 0.05). Blastocysts graded as AA showed a significantly higher euploidy rate than the others, which indicated that the Gardner morphology grade was an important characteristic for predicting euploid blastocysts.

**Table 2:** Relationship between Gardner grade and euploidy of blastocysts.

	Rates in different Gardner grades of blastocysts (%)				In total
	AA	AB or BA	BB	BC or CB	
Frozen in D5, n	175	257	274	67	773
Younger than 38, n	127	195	198	47	567
Euploid	98.43 (125/127)	68.21 (133/195)	50.00 (99/198)	38.30 (18/47)	66.14 (375/567)
Mosaic	0.00 (0/127)	11.79 (23/195)	15.15 (30/198)	8.51 (4/47)	10.05 (57/567)
Aneuploid	1.57 (2/127)	20.00 (39/195)	34.85 (69/198)	53.19 (25/47)	23.81 (135/567)
Equal or older than 38, n	48	62	76	20	206
Euploid	70.83 (34/48)	45.16 (28/62)	19.74 (15/76)	20.00 (4/20)	39.32 (81/206)
Mosaic	0.00 (0/48)	14.52 (9/62)	13.16 (10/76)	10.00 (2/20)	10.19 (21/206)
Aneuploid	29.17 (14/48)	40.32 (25/62)	67.11 (51/76)	70.00 (14/20)	50.49 (104/206)
Frozen in D6, n	74	170	256	123	623
Younger than 38, n	57	111	175	79	422
Euploid	96.49 (55/57)	53.15 (59/111)	38.29 (67/175)	41.77 (33/79)	50.71 (214/422)
Mosaic	0.00 (0/57)	10.81 (12/111)	14.29 (25/175)	8.86 (7/79)	10.43 (44/422)
Aneuploid	3.51 (2/57)	36.04 (40/111)	47.43 (83/175)	49.37 (39/79)	38.86 (164/422)
Equal or older than 38, n	17	59	81	44	201
Euploid	88.24 (15/17)	35.59 (21/59)	20.99 (17/81)	22.73 (10/44)	31.34 (63/201)
Mosaic	0.00 (0/17)	11.86 (7/59)	17.28 (14/81)	2.27 (1/44)	10.95 (22/201)
Aneuploid	11.76 (2/17)	52.54 (31/59)	61.73 (50/81)	75.00 (33/44)	57.71 (116/201)

### Relationship of morphokinetic characteristics and clinical parameters to euploidy of blastocysts

Morphokinetic characteristics of time to reach two cells (t2), three cells (t3), four cells (t4), five cells (t5), and time to reach blastocyst (tB) of blastocysts frozen on D6 were significantly delayed compared to blastocysts frozen on D5 (*P* < 0.05), and the KIDScore of D5 blastocysts was higher than that of D6 blastocysts (*P* < 0.05) (Table 3). In all blastocysts, Spearman correlation of euploidy to t2, t3, t4, t5, tB, KIDScore, female age, clinical indication of PGT-A, Gardner grade of blastocysts, and the number of embryonic frozen days are presented in Table 4. t2, t3, t4, t5, and tB, female age, and the number of embryonic frozen days were significantly and negatively correlated with euploidy in all blastocysts (*P* < 0.05), indicating that the possibility of euploidy increased as t2, t3, t4, t5, tB, female age, and the number of embryonic frozen days decreased. KIDScore and Gardner grades were significantly and positively correlated with the euploidy of blastocysts, suggesting that the possibility of euploidy increased as the KIDScore and Gardner grade increased (*P* < 0.05). In addition, there was no significant correlation between euploidy and the clinical indication for PGT-A (*P* > 0.05).

**Table 3:** Morphokinetic characteristics of blastocysts in this study.

	KIDScore	Time of morphokinetic parameters (hour, mean ± SD)				
		t2	t3	t4	t5	tB
Frozen in D5	8.03 ± 0.75	23.50 ± 0.75	34.33 ± 0.90	35.63 ± 1.32	45.84 ± 1.54	93.95 ± 1.94
Younger than 38	8.05 ± 0.75	23.51 ± 0.76	34.29 ± 0.90	35.56 ± 1.29	45.74 ± 1.56	93.87 ± 1.93
Equal or older than 38	7.97 ± 0.75	23.50 ± 0.72	34.43 ± 0.92	35.84 ± 1.38	46.09 ± 1.47	94.15 ± 1.97
Frozen in D6	6.42 ± 1.01	24.85 ± 1.53	36.12 ± 1.92	38.94 ± 3.64	48.27 ± 2.90	109.38 ± 3.96
Younger than 38	6.47 ± 1.05	24.73 ± 1.54	36.04 ± 1.95	38.81 ± 3.73	48.20 ± 2.94	109.18 ± 3.92
Equal or older than 38	6.32 ± 0.94	25.11 ± 1.48	36.31 ± 1.86	39.20 ± 3.45	48.40 ± 2.82	109.80 ± 4.02
In total	7.31 ± 1.19	24.11 ± 1.34	35.13 ± 1.70	37.11 ± 3.09	46.92 ± 2.55	100.83 ± 8.24

**Table 4:** Relationship between characteristics and euploid by Spearman correlation analysis.

Spearman correlation analysis	Morphokinetic and clinical parameters									
	t2	t3	t4	t5	tB	KIDScore	Female age	Clinical indication of PGT-A	Gardner grade	Frozen days
r	− 0.445	− 0.510	− 0.501	− 0.577	− 0.255	0.398	− 0.223	0.035	0.376	− 0.145
p	< 0.05	< 0.05	< 0.05	< 0.05	< 0.05	< 0.05	< 0.05	0.185	< 0.05	< 0.05

**Figure 1:**
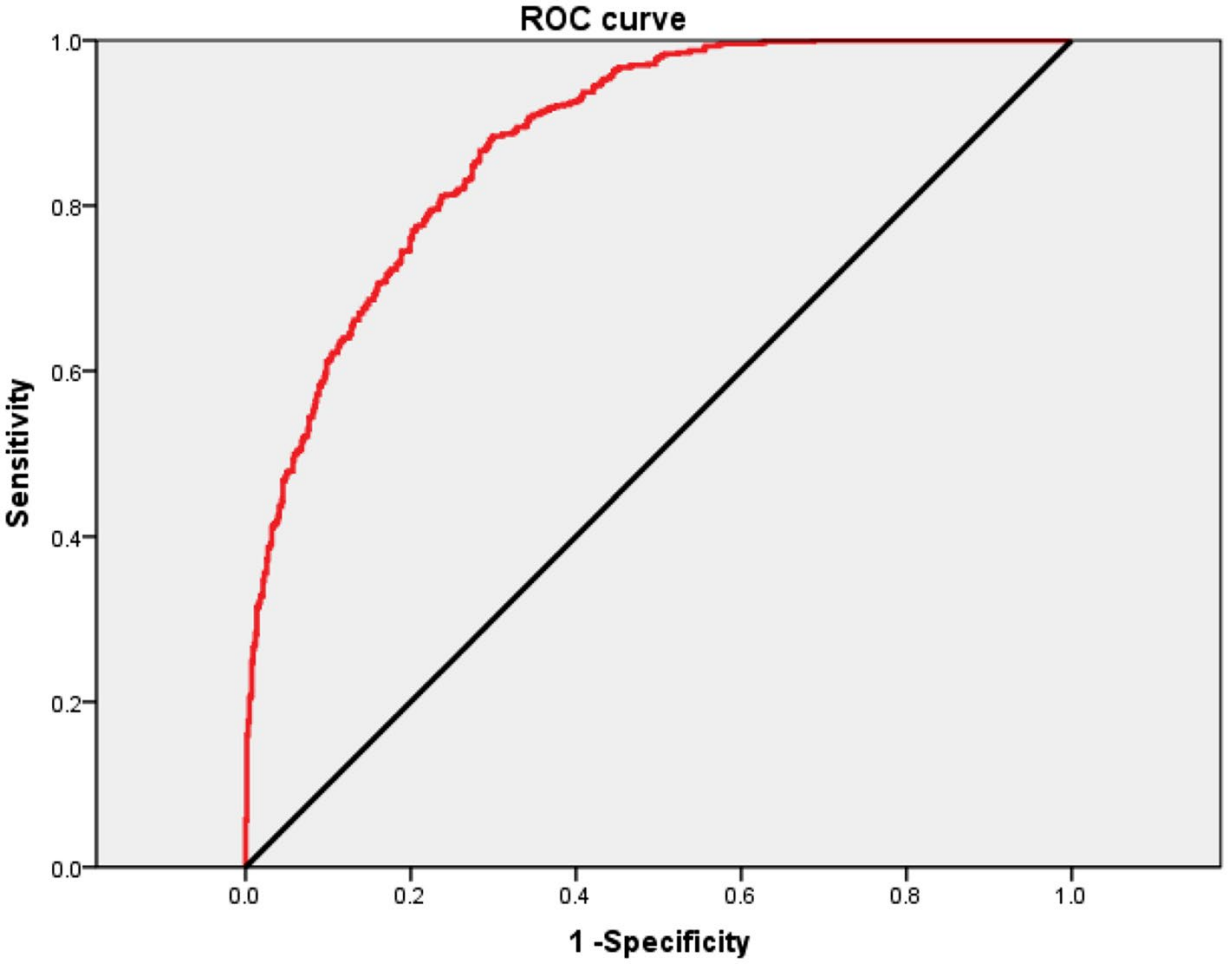
ROC of characteristics to predict euploidy of blatocysts. The ROC curve was showed in red and the black one was the reference line. X-axis showed the 1-specificity and Y-axis showed the sensitivity.

Two-logistic regression was used to analyse the relationship between euploidy and t2, t3, t4, t5, tB, KID score, female age, Gardner grade, and number of embryonic frozen days (Table 5). In all blastocysts, the result showed that t2 (OR 0.730, 95% CI 0.584–0.913), t3 (OR 0.597, 95% CI 0.460–0.775), t5 (OR 0.544, 95% CI 0.468–0.633), KIDScore (OR 0.544, 95% CI 0.389–0.762), female age (OR 0.892, 95% CI 0.865–0.920), Gardner grade (OR 1.790, 95% CI 1.436–2.231), and number of embryonic frozen days (OR 0.150, 95% CI 0.055–0.407) were significantly correlative to euploid (*P* < 0.05), but t4 (OR 1.005, 95% CI 0.846–1.195) and tB (OR 0.935, 95% CI 0.869–1.005) were not significantly correlative to euploidy (*P* > 0.05). Two-logistic regression was used to adjust OR and showed that t2 (adjusted OR 0.742, 95% CI 0.593–0.928), t3 (adjusted OR 0.601, 95% CI 0.489–0.738), t5 (adjusted OR 0.543, 95% CI 0.472–0.625), KIDScore (adjusted OR 0.646, 95% CI 0.488–0.855), female age (adjusted OR 0.891, 95% CI 0.864–0.919), Gardner grade (adjusted OR 1.728, 95% CI 1.392–2.145), and number of embryonic frozen days (adjusted OR 0.341, 95% CI 0.218–0.535) were significantly correlated with euploidy (*P* < 0.05). The predicted probability value of each blastocyst was also calculated by two-logistic regression. A receiver operating characteristic (ROC) curve was used for the area under the curve (AUC) among t2, t3, t5, KIDScore, female age, Gardner grade, and number of embryonic frozen days in the prediction of euploid blastocysts (Fig. 1). The ROC curve is shown in red, and the black curve is the reference line. The results represented that the model was effectively to predict euploidy of blastocysts (AUC = 0.879). The Youden index represented maximum when predicted probability value was 0.4182. At this point, the predictive cut-off value was 0.4182, sensitivity was 0.884, and specificity was 0.700 in this model. When predicted probability value of a blastocyst with euploid unknown was equal or greater than 0.4182, the blastocyst was predicted be euploid. The results showed that the model calculated by t2, t3, t5, KIDScore, female age, Gardner grade, and number of embryonic frozen days was capable of predicting the euploidy of blastocysts more effectively than a single parameter of t2 (AUC = 0.243), t3 (AUC = 0.206), t5 (AUC = 0.166), KIDScore (AUC = 0.730), female age (AUC = 0.371), Gardner grade (AUC = 0.707), and number of embryonic frozen days (AUC = 0.572) (Table 5).

**Table 5:** Relationship between characteristics and euploid by two-logistic regression and ROC curve analysis.

Two-logistic regression analysis	OR	95% CI	P	Adjusted OR	95% CI	P	AUC of ROC curve
t2	0.730	0.584–0.913	< 0.05	0.742	0.593–0.928	< 0.05	0.243
t3	0.597	0.460–0.775	< 0.05	0.601	0.489–0.738	< 0.05	0.206
t4	1.005	0.846–1.195	0.954	–	–	–	–
t5	0.544	0.468–0.633	< 0.05	0.543	0.472–0.625	< 0.05	0.166
tB	0.935	0.869–1.005	0.069	–	–	–	–
KIDScore	0.544	0.389–0.762	< 0.05	0.646	0.488–0.855	< 0.05	0.730
Female age	0.892	0.865–0.920	< 0.05	0.891	0.864–0.919	< 0.05	0.371
Gardner grade	1.790	1.436–2.231	< 0.05	1.728	1.392–2.145	< 0.05	0.707
Frozen days	0.150	0.055–0.407	< 0.05	0.341	0.218–0.535	< 0.05	0.572

### Relationship between morphokinetic characteristics, morphological characteristics, and clinical parameters to live birth

A total of 295 euploid blastocysts, including 186 frozen on D5 and 109 frozen on D6, were transferred in 295 FET treatments. The intrauterine pregnancy rate (80.11% vs. 72.48%) and live birth rate (65.59% vs. 58.72%) of transferred D5 blastocysts were higher, but not significantly, than those of D6 blastocysts (*P* > 0.05) (Table 6). The non-pregnancy rate (19.89% vs. 27.52%) of D5 blastocysts transferred was lower, but not significantly, than that of D6 blastocysts (*P* > 0.05). The abortion rate (18.12% vs. 18.99%) was nearly the same in D5 and D6 blastocysts (*P* > 0.05). Live birth was not significantly correlated with t3, t4, t5, tB, KIDScore, female age, clinical indication of PGT-A, Gardner grade of blastocysts, endometrial thickness, or number of embryonic frozen days (Table 7) (*P* > 0.05). t2 was significantly correlated with live births (*P* < 0.05). However, live birth was not significantly correlated with t2 by two-logistic regression (OR 0.856, 95% CI 0.602–1.219, and *P* > 0.05). ROC was also used for t2 to predict live births, and AUC = 0.569. The results showed that t2, t3, t4, t5, tB, KIDScore, female age, clinical indication for PGT-A, Gardner grade, endometrial thickness, and number of embryonic frozen days were not capable of predicting live birth after FET in PGT-A treatments.

**Table 6:** FET outcomes after euploid blastocysts transferred in this study.

	Number of FET	Non pregnant rate (%)	Intrauterine pregnancy rate (%)	Abortion rate (%)	Live birth rate (%)
Frozen in D5	186	19.89 (37/186)	80.11 (149/186)	18.12 (27/149)	65.59 (122/186)
AA	63	20.63 (13/63)	79.37 (50/63)	16.00 (8/50)	66.67 (42/63)
AB or BA	67	17.91 (12/67)	82.09 (55/67)	21.82 (12/55)	64.18 (43/67)
BB	46	17.39 (8/46)	82.61 (38/46)	15.79 (6/38)	69.57 (32/46)
BC or CB	10	40.00 (4/10)	60.00 (6/10)	16.67 (1/6)	50.00 (5/10)
Frozen in D6	109	27.52 (30/109)	72.48 (79/109)	18.99 (15/79)	58.72 (64/109)
AA	29	20.69 (6/29)	79.31 (23/29)	21.74 (5/23)	62.07 (18/29)
AB or BA	41	31.71 (13/41)	68.29 (28/41)	21.43 (6/28)	53.66 (22/41)
BB	29	20.69 (6/29)	79.31 (23/29)	17.39 (4/23)	65.52 (19/29)
BC or CB	10	50.00 (5/10)	50.00 (5/10)	0.00 (0/5)	50.00 (5/10)
In total	295	22.71 (67/295)	77.29 (228/295)	18.42 (42/228)	63.05 (186/295)

**Table 7:** Relationship between characteristics and live birth by Spearman correlation analysis.

	Frozen days	Female age	Clinical indication of PGT-A	Gardner grade	Endometrial thickness	t2	t3	t4	t5	tB	KID score
r	0.069	− 0.089	0.055	0.019	0.038	− 0.115	− 0.065	− 0.027	− 0.021	− 0.044	0.059
p	0.239	0.129	0.342	0.741	0.516	< 0.05	0.267	0.640	0.722	0.452	0.311

## Discussion

In the current study, we failed to predict live births using the D5 KIDScore v3.1 model or clinical characteristics in PGT-A treatments, which was inconsistent with previous research results^12^. In fact, we found that live birth performance was not significantly related to morphology, morphokinetic characteristics, or clinical parameters after euploid blastocyst FET, although we set strict limits to minimise individual variation between patients. A model that can effectively predict FET outcomes using the basis and objective parameters would certainly be desirable. However, we believe that euploid embryos are vital to FET outcomes and good morphokinetic or morphological performance most likely indicates euploid embryos. The positive results of previous studies proved that embryos with high developmental potency were screened for FET. However, the predictive power and significance diminished as pregnancy progressed. We considered that many individual factors, such as endometrial conditions, may affect the prediction of FET outcomes. For example, quite a few patients with RIF suffered from window of implantation shifting, which could cause failure even in FET outcomes of euploidy^19,20^. Therefore, although previous studies have demonstrated that good morphokinetic performance of embryos is capable of predicting implantation, pregnancy, or live birth, we suspected that if it was the prediction of FET outcomes, more individual parameters of patients rather than morphokinetic characteristics of embryos should be considered to evaluate the prediction effects. Indeed, in the present study, we proved that morphokinetic characteristics of embryos were not significantly related to live birth when euploid blastocysts were transferred. We believe that it is more rigorous and objective to create a model using morphokinetic, morphological characteristics, or clinical parameters to predict the euploidy of blastocysts, rather than to predict the FET outcomes such as pregnancy or live birth, which could be more likely to be affected by individual factors.

Here, we used a model with a combination of t2, t3, t5, KIDScore, Gardner grade, female age, and number of embryonic frozen days to effectively predict the euploidy of blastocysts (AUC = 0.879, sensitivity = 0.884, and specificity = 0.700). We have summarised the criteria for blastocyst selection. In IVF treatments with non-PGT, we collected t2, t3, and t5, KIDScore, Gardner grade, and number of embryonic frozen days of blastocysts from TLM, female patient age, and input these into our model. Then, two-logistic regression was used to analyse the predicted probability values of blastocysts. When the predicted probability value was equal or larger than 0.4182, the blastocysts were predicted to be euploid. Quality assurance (QA) was guaranteed by two embryologists during blastocyst development. However, more samples should be included in future studies to prove the effectiveness of the model and to further optimise it. It is also worth noting that KIDScore was not a valid predictor of euploidy in the current study. The KIDScore is a comprehensive judgment based on the characteristics of each embryo during development. Some embryos with poor morphokinetic characteristics in the early stages acquired high morphological grades, resulting in similar or even identical scores to well-developed embryos with average morphological grades. In the present study, the combined characteristics model performed better than the KIDScore algorithm (AUC = 0.879 vs. AUC = 0.730, respectively). Therefore, more morphokinetic characteristics must be included in the predictive model.

Euploidy is an important factor affecting the developmental potency of embryos. In IVF treatments, the aneuploidy rate of 2PN embryos is 25–40%, and the rate of aneuploid embryos increases with female age^21,22^. PGT-A can help avoid aneuploid embryos from FET; however, PGT-A is a limiting medical technology in IVF treatments in China, and only patients who strictly conform to clinical indications, including FAA, RIF, RPL, and STS, can receive PGT-A treatment. Euploid embryos were selected for FET using PGT-A in patients with FAA, which increased the cumulative pregnancy rate^22^. Patients with RIF and RPL suffered from multiple persistent pregnancy failures and using PGT-A could decrease the abortion rate and increase the pregnancy rate in patients^23,24^. STS is one of the clinical indications of PGT-A, since the aneuploidy rate of sperm from patients diagnosed with STS increases, resulting in a higher aneuploidy rate in embryos than in patients without STS^25^. During the development period of embryos of PGT-A treatments in the present study, we found that the clinical parameters, morphokinetics, and morphological characteristics of euploid embryos are worthy of attention and reference, which could aid in the selection of euploid embryos in non-PGT IVF treatments.

The mosaic rate of blastocysts of up to 26% was acceptable in PGT-A treatments, and it was controversial whether mosaic embryos could be transferred^26,27^. Previous study demonstrated that many blastocysts diagnosed as mosaic were caused by the amplification method in PGT-A, technical reasons for biopsy, and poor embryo quality^27^. In fact, many blastocysts diagnosed as mosaic have been proven to be euploid after FET. Embryos with low mosaic levels are recommended for FET when euploid embryos are lacking^28^. However, FET of mosaic embryos is not common in China, and the mosaic is the same as aneuploidy in embryo selection for FET in most IVF clinics. In PGT-A treatments, blastocysts with good development potency were avoided for FET because they were diagnosed as mosaics. More studies on the FET outcomes of mosaic embryos are needed.

In this study, we showed our research result of predictive effect of female age, clinical indications of PGT, number of embryonic frozen days, morphological characteristics, morphokinetic characteristics on the euploid blastocysts and live birth, which may differ from IVF clinics^9^. We did our own research in IVF lab, and we found that the model we built was effective to predict the euploidy of blastocysts, but failed to predict live birth after FET. However, the present study had some limitations. Since this was a retrospective study including 1396 blastocysts from a single IVF clinic, more samples from different clinics are required to further optimise this model. A prospective randomised study is needed to confirm the relevance of morphokinetics, morphological characteristics, and clinical parameters in euploid blastocysts. Although rigorous QA and statistical analysis were performed in previous studies, and AI-based model decided the euploidy of blastocysts by a mathematical method, the adjusted parameters of embryos from embryologists were subjective, which were difficult to avoid. AI-based model could quantify the developmental potency of embryos, but the reality was more complicated than that of the model. Here, the prediction effects of this model were only referenced by embryologists, although it worked well. Our study provided non-PGT IVF treatments with a method to predict the euploid blastocysts noninvasively, but more samples are required to verify the effectiveness of this model.

## Methods

### Patients

This study used a total of 403 patients with RIF (n = 83), RPL (n = 184), STS (n = 83), and FAA (n = 53) who received PGT-A treatment at the Reproductive Medicine Centre of Xuzhou Maternal and Child Health Care Hospital from January 2019 to January 2022. None of the patients had chromosomal karyotype abnormalities. This study was approved by the Ethics Committee of Reproductive Medicine of Xuzhou Maternal and Child Health Care Hospital, and the patients received detailed genetic counselling and signed relevant informed consent forms.

### Procedure

All methods were performed in accordance with the relevant guidelines and regulations. Controlled ovarian hyperstimulation (COH) was performed using long-acting or antagonist protocols. Human chorionic gonadotropin (HCG) was injected when the largest diameter of the follicles reached 18 mm or the diameter of two follicles reached 16 mm. Transvaginal ovum pick-up (OPU) was guided by b-ultrasound. Intracytoplasmic sperm injection (ICSI) was performed for all the PGT-A treatments. Embryonic culture and morphological observations were performed using the G-TL culture system (Vitrolife, Sweden) and TLM system (Vitrolife). During embryo development, TLM provided parameters of time to reach t2, t3, t4, t5, and tB by D5 KIDScore v3.1 model. To maintain QA, two embryologists adjusted these parameters after 5–6 days of embryonic culture post fertilisation, and morphological grading of blastocysts was performed (referring to the Gardner blastocyst grading standard) by D5 KIDScore v3.1 model, where two embryologists adjusted the grades. A biopsy of blastocysts graded above BC or CB was performed. PGT-A was used for the genetic testing of biopsy products. Euploid blastocysts were prepared for FET according to the PGT results. Clinical pregnancy was defined as blood HCG > 20 mIU/mL 14 days after transfer. Intrauterine pregnancy was defined as gestational sac (GS) detected by b-ultrasound 28 days after blastocyst transfer. At 16–20 weeks of gestation, amniocentesis was performed to analyse foetal chromosome euploidy and karyotypes. Follow-up visits were performed until the end of the pregnancy or live birth.

### Statistical analysis

SPSS 19.0 software (SPSS Inc., Chicago, IL, USA) was used for data statistics. The Student’s t-test was used to analyse the differences in female age, KIDScore, t2, t3, t4, t5, and tB among the groups. Statistical significance was set at *P* < 0.05. The chi-square test was used to analyse the differences in euploidy, mosaic, aneuploidy, non-pregnancy, intrauterine pregnancy, abortion, and live birth rates among the groups. Spearman’s correlation analysis was used to analyse the relationship between the characteristics or parameters and euploidy. ROC curve and AUC were used to evaluate the predictive effectiveness of euploid to characteristics or parameters.

## Data Availability

The data that support the findings of this study are available from the corresponding author.
